# Hematinic Potential of Olive Leaf Extract: Evidence from an In Vivo Study in Mice and a Pilot Study in Healthy Human Volunteers

**DOI:** 10.3390/nu15194095

**Published:** 2023-09-22

**Authors:** Shinji Kondo, Farhana Ferdousi, Jinchang Zhao, Sofya Suidasari, Miki Yokozawa, Ken Yamauchi, Ken-ichi Tominaga, Hiroko Isoda

**Affiliations:** 1 Alliance for Research on the Mediterranean and North Africa (ARENA), University of Tsukuba, Tsukuba 305-8572, Japan; 2 Faculty of Life and Environmental Sciences, University of Tsukuba, Tsukuba 305-8575, Japan; 3 Graduate School of Life and Environmental Science, University of Tsukuba, Tsukuba 305-8572, Japan; 4 Nutrition Act Co., Ltd., Ginza, Tokyo 104-0061, Japan; 5 Open Innovation Laboratory for Food and Medicinal Resource Engineering (FoodMed-OIL), National Institute of Advanced Industrial Science and Technology (AIST), Tsukuba 305-8577, Japan

**Keywords:** olive leaf extract, erythropoiesis, reticulocyte, iron homeostasis, anemia, pilot study

## Abstract

Natural resources have recently received considerable attention as complementary or alternative hematinic agents. In this regard, olive leaf extract, which is rich in bioactive phenolic compounds, has been reported to induce erythroid differentiation in human hematopoietic stem cells. Therefore, in the present study, we aimed to explore the potential hematinic properties of aqueous olive leaf extract (WOL) in vivo. After 24 days of administering WOL to healthy mice orally, red blood cell (RBC), hematocrit, reticulocyte, and reticulocyte hemoglobin content (CHr) showed a significant increase. Additionally, WOL promoted plasma iron levels and the expression of splenic ferroportin (Fpn), an iron transporter. Additionally, a single-arm pilot study involving a limited number of healthy volunteers was conducted to assess WOL’s feasibility, compliance, and potential benefits. Following an 8-week intervention with WOL, RBC count and hemoglobin level were significantly increased. Notably, there were no significant changes in the safety measures related to liver and kidney functions. Furthermore, we identified oleuropein and oleuroside as the active components in WOL to induce erythroid differentiation in the K562 cell line. Altogether, our study presents evidence of the hematinic potential of WOL in the in vivo studies, opening up exciting possibilities for future applications in preventing or treating anemia.

## 1. Introduction

Anemia refers to a reduction in either the total number of red blood cells (RBCs) or the blood’s ability to carry oxygen. It stands as the most prevalent blood disorder, affecting 40% of children aged 6–59 months, 37% of pregnant women, and 30% of women aged 15–49 years globally in 2019, according to the World Health Organization (WHO) [1]. There are many types of anemia, which can be caused by blood loss, the insufficient production of RBCs, or chronic disease. Among these, iron deficiency anemia is the most common and is reported to be particularly common in young adults and women [2]. Anemia can lead to extreme fatigue, weakness, dizziness, shortness of breath, and many other symptoms.

Erythropoiesis is the process through which RBCs are produced in the bone marrow. It involves several stages: initially, hematopoietic stem cells (HSCs) differentiate into specialized erythroid progenitors known as burst-forming unit-erythroid (BFU-E) and colony-forming unit-erythroid (CFU-E). Subsequently, nucleated precursors transform into various forms of erythroblasts, such as basophilic, polychromatophilic, and orthochromatic erythroblasts, while accumulating hemoglobin and undergoing nuclear condensation. Finally, reticulocytes mature into fully formed erythrocytes, which are then released into the bloodstream [3]. The first stage demands erythropoietin, while the second stage requires iron. Ineffective erythropoiesis may occur when erythroid progenitor precursors do not mature properly, die during transformation into erythrocytes, or develop into abnormal RBCs. This ultimately leads to a decrease in the number of erythrocytes, resulting in anemia [4]. Therefore, the primary goals for anemia treatment are to improve defective erythropoiesis and increase the production of erythrocytes.

Currently available treatments for anemia include supplementation with iron and other nutrients, blood transfusions, and erythropoiesis-stimulating agents (ESAs), each aimed at increasing RBC count [5]. However, absorption by the human body is limited, and conventional therapies are associated with side effects. For example, blood transfusions can cause allergies and immunomodulatory effects [6], and excessive iron intake can cause hemochromatosis [7]. Thus, there remains an unmet medical need for new therapeutic compounds for the treatment of anemia.

In recent years, natural resources have received significant attention as complementary or alternative treatments for anemia. Leaves of *Trigonella foenum-graecum*, commonly known as Fenugreek, are rich in iron and folate and have been reported to promote RBC production [8,9]. *Psidium guajava* is a popular medicinal plant in India, and its fruits and leaves have been shown to have an active effect on hemoglobin (HGB) levels in clinical trials [10,11]. It can be used to treat anemia because of its phenolic and alkaloid compounds [12]. Black pepper and its primary metabolite, piperine, can inhibit hepcidin overexpression and prevent the pathogenesis of inflammatory anemia [13]. Luteolin, one of the active constituents of olive, has been reported to alleviate renal anemia in mice by suppressing the PHD2/HIF-2α axis and oxidative stress [14].

In this regard, olive leaf extract (OLE), rich in bioactive phenolic compounds, is widely used as a food supplement and drug for its various benefits. OLE has been reported to exhibit anti-inflammatory, anti-cancer, anti-diabetic, and neuroprotective effects [15,16,17]. In our previous clinical trial study, we found that the long-term consumption of olive-leaf tea had beneficial effects on hematological parameters [18]. Additionally, OLE has been reported to induce monocyte/macrophage differentiation [19], while its component apigenin 7-*O*-beta-D-glucoside, also known as apigetrin, induces erythroid differentiation in the human chronic myeloid leukemia cell line K562 [20]. On the other hand, two other components of OLE, namely apigenin 7-glucoside (A7G) and luteolin 7-glucoside (L7G), have been reported to induce human hematopoietic stem cells (hHSCs) differentiation into the erythroid lineage [21]. Furthermore, in hHSCs, the aqueous extract of olive leaves (WOL) induced erythroid differentiation and contributed to oxygen and iron homeostasis, heme metabolism, and hemoglobin biosynthesis [22].

The primary objective of this study was to investigate the hematinic properties of WOL in mice. Additionally, we conducted a single-arm pilot study with a limited number of healthy volunteers to assess the feasibility, compliance, and potential benefits of WOL supplementation. Furthermore, we sought to identify the active hematinic components present in WOL. To achieve this, we studied the effects of WOL components on inducing erythroid differentiation in the K562 cell line, an undifferentiated progenitor cell [23] expressing erythroid markers such as glycophorin A (GYPA) [24].

## 2. Materials and Methods

### 2.1. WOL Extraction Procedure

Olive leaves were collected from a farm in Tunisia in mid-December 2018 as described in a previous study [22]. These leaves were naturally air-dried in the shade at a temperature ranging from 20 to 25 °C. The dried leaves were then processed into powder form using a food processor. This powdered leaf material was subsequently subjected to extraction using sterile distilled hot water for a duration of 1 h. The resulting extract was then filtered through diatomite to eliminate foreign particles and was then concentrated. After sterilization, dextrin was introduced as an excipient, and freeze-drying was conducted. In the final step, the extracted powder was ground and passed through a 40-mesh sieve before being stored in a warehouse at 15 °C. To produce WOL capsules, the primary ingredient, OLE powder, was mixed with excipients and then encapsulated into hydroxypropyl methylcellulose (or Hypromellose; HPMC) hard capsules.

### 2.2. Animal Experimental Design

The animal experiments were carried out in accordance with the Regulation for Animal Experiments in the University of Tsukuba and the Fundamental Guideline for Proper Conduct of Animal Experiments and Related Activities in Academic Research Institutions under the jurisdiction of the Ministry of Education, Culture, Sports, Science and Technology of Japan.

Male ICR mice of four weeks of age were purchased from Charles River, Japan. The mice were housed under standard pathogen-free conditions with 12 h light/dark schedules (light time 7 a.m. to 7 p.m.) at 22 ± 2 °C and relative room humidity 60 ± 10%. All mice were housed individually and given free access to food and drinking water. After acclimatization for one week, the mice were used for the experiments.

**Figure 1:**
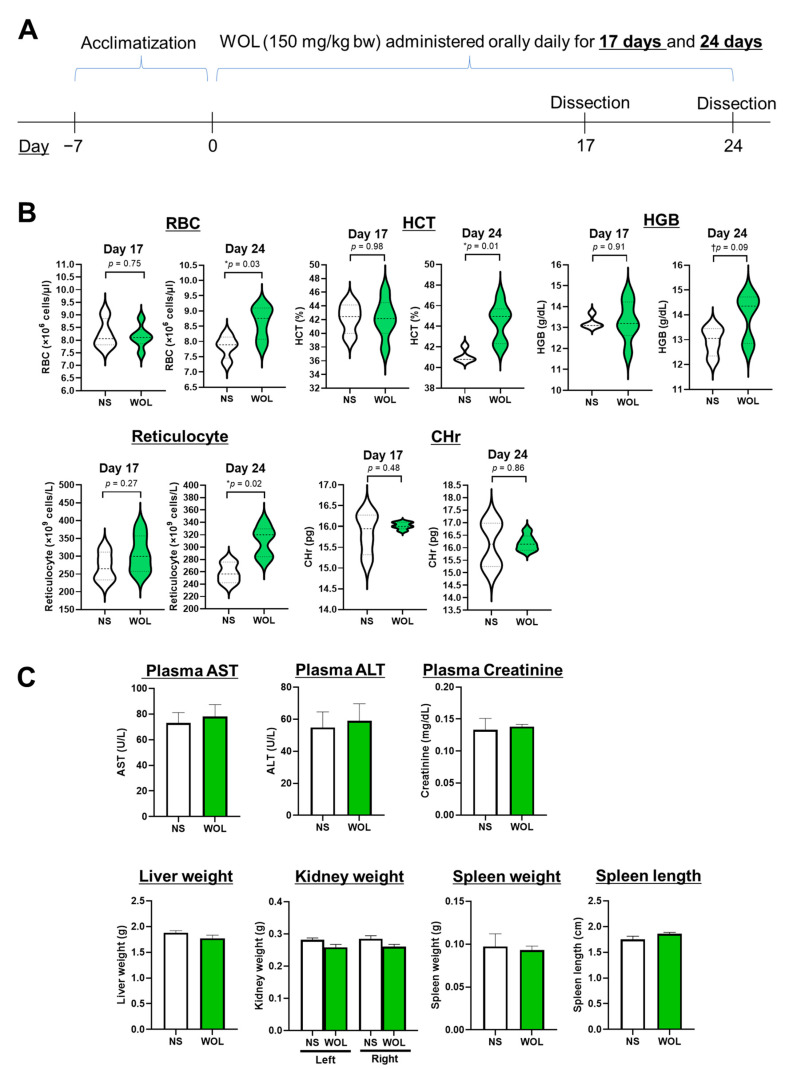
WOL showed beneficial effects on hematological parameters without toxicity in mice. (**A**) The mice were orally administrated with 150 mg/kg bw WOL for 17 or 24 days. (**B**) Hematological parameters (RBC, HCT, HBG, Reticulocyte, and CHr). (**C**) Safety evaluation of WOL after 24 days of oral intake. Plasma AST and ALT are hepatic dysfunction markers, and plasma creatinine is a renal dysfunction marker. Each value represents the mean ± SE for *n* = 4 (NS) and *n* = 6 (WOL) in each group. Significant difference from NS group at ^†^ *p* < 0.1, * *p* < 0.05 by unpaired two-tailed Student’s *t*-test. The lines in the middle of the violin chart area refer to the mean value. NS: normal saline-treated control group; WOL: aqueous extract of olive leaves-treated group; RBC: red blood cell; HCT: hematocrit; HGB: hemoglobin; CHr: reticulocyte hemoglobin content; AST: aspartate aminotransferase; ALT: alanine aminotransferase.

Twenty mice were divided randomly into two sets: a normal saline group (NS group, *n* = 8) and a WOL group (*n* = 12). In the WOL group, the mice were given WOL orally at 150 mg/kg body weight each day, while the control group received an equal volume of normal saline (NS) daily. The dosage intended for mice administration was established through a preliminary investigation. In the preliminary phase, the mice were subjected to hematological testing after being given 50, 150, and 250 mg/kg bw WOL. The outcomes exhibited a marked inclination towards elevated HGB and HCT levels when the doses exceeded 150 mg/kg bw. Consequently, for the current study, the WOL dosage was determined as 150 mg/kg bw. After 17 days of treatment, half of the mice from each group were euthanized and examined, and the remaining half were studied after 24 days of treatment. The experimental schedule is shown in Figure 1A.

To begin, the mice were administered isoflurane anesthesia, and blood samples were obtained from them while under anesthesia. Subsequently, the mice were euthanized through cervical dislocation. A portion of the collected blood was processed into plasma for subsequent biochemical tests. The mice were then positioned on sterile surgical pads with their abdomens facing upward. The skin in the abdominal and hindlimb regions was sterilized by applying 70% ethanol. Next, the abdominal cavity was incised using dissecting scissors. Organs, including the liver, spleen, both kidneys, and the intestine, were carefully extracted and rinsed with 10% PBS. After the washing procedure, the weight and length of the organs were recorded, and they were promptly frozen in liquid nitrogen for storage at −80 °C until use.

### 2.3. Hematological and Biochemical Tests

Blood was drawn from the inferior vena cava and placed into tubes containing heparin. Plasma was then obtained by subjecting the samples to centrifugation at 1000× *g* for 15 min at a temperature of 4 °C. The hematological parameters examined in this study included RBC count, hematocrit (HCT), hemoglobin (HGB) levels, immature RBCs (reticulocytes), and reticulocyte hemoglobin content (CHr). Additionally, we assessed the levels of iron, markers of hepatic dysfunction (aspartate aminotransferase (AST) and alanine aminotransferase (ALT)), as well as a marker of renal dysfunction (creatinine) in the plasma. All the blood and plasma analyses were conducted at the BioSafety Research Center in Japan.

### 2.4. RNA Extraction and Real-Time PCR

Total RNA samples from mouse tissues and K562 cells were isolated utilizing the RNA isolation reagent ISOGEN (Nippon Gene, Tokyo, Japan), following the guidelines provided by the manufacturer. The concentration of total RNA was determined using a NanoDrop 2000 spectrophotometer (ThermoFisher Scientific, Waltham, MA, USA). Gene expression was assessed using TaqMan probes (ThermoFisher Scientific). cDNA solutions were prepared using superscript IV VILO master mix (ThermoFisher Scientific) according to the manufacturer’s instructions. TaqMan real-time RT-PCR amplification reactions were carried out using an Applied Biosystems 7500 Fast Real-Time System (ThermoFisher Scientific) to quantify the mRNA levels. All primer sets and the TaqMan Universal PCR Master Mix were procured from ThermoFisher Scientific. The primers used for the mouse tissues were as follows: ferroportin (*Fpn*; Mm00489837_m1), hepcidin (*Hamp*; Mm04231240_s1), hypoxia-inducible factor 1 alpha (*Hif1a*; Mm00468869_m1), and erythropoietin (*Epo*; Mm01202755_m1). Actin Beta (*Actb*; Mm02619580_g1) was used as an internal control. The primers for *GYPA* (Hs01068079_s1) were used for K562 with *ACTB* (Hs03023880_g1) as the internal control.

### 2.5. Pilot Study

In order to assess the feasibility, compliance, and potential benefits of OLE, a pilot study was conducted on healthy volunteers. The study included Japanese female participants between the ages of 20 and 50 who provided informed consent.

The exclusion criteria were:

1. Currently undergoing treatment with any form of drug or herbal medicine (except as needed).
2. History of severe liver, heart, kidney, gastrointestinal, respiratory, endocrine, or metabolic diseases (appendicitis is acceptable).
3. Allergic reactions to the test food after oral intake.
4. History of treatment for heart failure, myocardial infarction, or similar conditions.
5. Currently taking ‘health foods’ (the term ‘health foods’ encompasses all dietary supplements, including but not limited to those labeled as “Foods with functional claims”, “Foods with nutrient function claims”, or “Foods for specified health uses”, which have been approved under the Food Labeling Act by the Ministry of Health, Labor, and Welfare in Japan).
6. Current or past history of drug allergies or food allergies.
7. Participation in other clinical trials within one month before obtaining consent or currently participating in another trial.
8. Currently pregnant, breastfeeding, or intending to become pregnant.
9. Heavy alcohol consumption.
10. Smoking habit.
11. History of experiencing negative mood or physical condition deterioration due to blood sampling in the past.
12. Likely to undergo lifestyle habit changes during the test period, such as long-term travel or others.
13. In addition, individuals deemed unsuitable for participation in this study by the principal investigator were also excluded.

A total of eight volunteers underwent the initial screening process for the study. One of them was found to be taking a supplement and, therefore, was deemed ineligible to participate in the study. Finally, seven volunteers (age range 29–47 years) were invited to participate in the study.

The study participants were administered 500 mg of OLE, divided into four capsules, once daily after meals in the morning for a duration of 8 weeks. The dosage selected for the human intervention study was set at 500 mg, which represents the highest standard dosage for a food supplement with a potential functional claim. Detailed information about the capsules is provided in Supplementary Table S1. The capsules were supplied by Nutrition Act Co., Ltd., Tokyo, Japan. Compliance with capsule ingestion was monitored during nutrition consultations, and the participants were given a capsule calendar at the start of the trial, which they submitted upon completion of the 8-week period. Throughout the intervention, the participants were requested to maintain their regular diet and physical activities without any imposed restrictions. Moreover, the participants were asked to report regarding the palatability of the supplement and any adverse effects experienced during the supplementation period.

Routine blood tests, including RBC count, HGB (%), serum Fe, and serum ferritin, along with other regular parameters, were tested at baseline and after 6 weeks and 8 weeks of supplementation. Furthermore, in order to evaluate the safety of the supplementation, any alterations in the parameters of liver function tests and renal function tests in response to OLE were also examined before and after the 8-week supplementation period. The liver function tests included serum levels of AST, ALT, and alkaline phosphatase (ALP). The renal function tests were serum creatinine and urine urea nitrogen.

### 2.6. HPLC Analysis

The HPLC analysis for apigenin (A), luteolin (L), hydroxytyrosol (HT), oleuropein (OP), A7G, and L7G was carried out using a Shimadzu Prominence LC-20 system as previously reported [22]. The HPLC column was ZORBAX SB-C18 (5 µm, φ 4.6 × 150 mm). Separation was carried out at 40 °C with a gradient elution program at a flow rate of 1.0 mL/min. The mobile phases were 10% formic acid in water (A) and a 1:1 mixture of acetonitrile and methanol (B). The gradient program was 0–100% (B) for 40 min, followed by a re-equilibration duration of 10 min. The monitoring wavelength was configured at 280 nm for A, L, HT, and OP, while for A7G and L7G, it was set at 331 nm. The injection volume of the WOL (50 mg/mL MeOH, 0.22 µm filtration) in the HPLC system was 10 µL.

For apigenin-7-*O*-rutinoside (A7R) and oleuroside (OS), the analysis condition was modified somewhat. The HPLC column was a TSKgel ODS-100 V (3 µm, φ2 × 150 mm). Separation was carried out at 40 °C with a gradient elution program at a flow rate of 0.2 mL/min. The mobile phases were 0.5% acetic acid (A) and acetonitrile (B). The following multistep linear gradient was applied: 0 min, 5% B; 5 min, 15% B; 25 min, 30% B; 35 min, 95% B. The monitoring wavelength was 311 nm for OS and 254 nm for A7R.

### 2.7. Cell Culture and WOL Treatment

The human chronic leukemia cell line K562 was obtained from the Riken Cell Bank (RCB0027). The cell line was maintained in Roswell Park Memorial Institute (RPMI) 1640 medium (Gibco) supplemented with heat-inactivated 10% fetal bovine serum (Gibco) and 1% antibiotic (100 U/mL of penicillin) (Gibco). The cells in the medium were maintained in a humidified incubator at 37 °C with 5% CO_2_. The cells were seeded at 1 × 10^5^ cells/mL density and passaged every two days. After two weeks of cell culture, the K562 cells were used for experiments. The K562 cells were seeded at a density of 2 × 10^4^ cells/mL in 6-well plates. After 24 h, the cells were treated with 120 µg/mL WOL and its eight components— A, A7G, A7R, HT, luteolin L, L7G, OP, and OS. The concentration of WOL used for treating the K562 cells (120 µg/mL) was established based on the concentration utilized in a previous study that investigated the induction of erythroid differentiation [22]. The treatment concentration of these eight components was decided based on their respective dry weight in 120 µg/mL WOL (OP: 35.36 µM, OS: 5.23 µM, HT: 2.79 µM, A: 0.31 µM, A7G: 0.49 µM, A7R: 2.26 µM, L: 0.66 µM, and L7G: 8.19 µM). Apigenin (50 mM) was used as a positive control (PC). In addition, co-treatment with an 8-component mixture, a 7-component combination (excluding OS), a 3-component mixture (comprising OP, OS, and HT), and a 2-component combination (involving OS and other components) were conducted to identify the active components. The medium was changed on the third day of treatment. After a total treatment period of 6 days, the cells were used to analyze gene expression, viability, and proliferation.

### 2.8. Flow Cytometry

The cluster of differentiation 235a (CD235a, also known as GYPA) antibody was tested by flow cytometric analysis of erythrocytes in the K562 cell. The cells were washed with 10% PBS and incubated with anti-GYPA antibody in PBS containing 1% bovine serum albumin for 30 min on ice. Then, they were washed with PBS and stained with the secondary antibody, FITC-conjugated goat anti-mouse IgG (MBL), in PBS containing 1% bovine serum albumin for 30 min. The results were analyzed on Guava Express (Luminex Corporation, Austin, TX, USA).

To assess viability, a volume of 20 μL of medium containing K562 cells treated with WOL or its individual components within a 6-well plate was transferred to a round-bottom 96-well plate. Subsequently, the cells were suspended in 180 μL of Guava ViaCount reagent (Luminex Corporation) and the results were analyzed with Guava ViaCount software.

### 2.9. MTT Assay

Cell proliferation was assessed through the employment of the MTT (3-(4,5-dimethylthiazol-2-yl)-2,5-diphenyltetrazolium bromide) assay. In this process, 50 μL of medium containing K562 cells treated with WOL or its individual components within a 6-well plate was transferred to 96-well plates. Subsequently, the cell concentration was subjected to a 2-fold dilution by adding an additional 50 µL of fresh medium. A solution of 5 mg/mL MTT, dissolved in phosphate-buffered saline, was introduced (10 μL per well) and allowed to incubate overnight. Following this, a 10% *w*/*v* sodium dodecyl sulfate solution (100 μL per well) was introduced and similarly incubated overnight. The resulting formazan formation was quantified using spectrophotometric analysis at 570 nm, utilizing a microplate reader designed for 96-well plates.

### 2.10. StatisticalAanalysis

An unpaired two-tailed Student’s *t*-test was used to compare the two groups. Data are represented as the mean ± standard error of the mean (SEM) unless otherwise mentioned.

In the pilot study, a one-way repeated measures ANOVA followed by Fisher’s least significant difference (LSD) test was employed to analyze pairwise differences in hematological parameters at 6 weeks and 8 weeks. A one-tailed paired *t*-test was used to conduct a pre–post evaluation of LFTs and RFTs.

The Shapiro–Wilk normality test and Levene’s tests were applied to all parameters in order to assess their distribution and homogeneity. A *p*-value < 0.05 was considered significant. Statistical analyses were performed with GraphPad Prism version 8.0 (GraphPad Software, Inc., Boston, MA, USA) and SPSS IBM SPSS Statistics version 28 (IBM Corp., Armonk, NY, USA).

## 3. Results

### 3.1. Animal Study

#### 3.1.1. WOL Exerted a Beneficial Impact on Hematological Parameters without Any Toxicity in Mice

To investigate the effects of WOL on erythropoiesis in mice, several hematological parameters were assessed. No significant difference was observed in the parameters after 17 days of WOL treatment. However, 24 days of oral administration of WOL resulted in significant increases in RBC count, hematocrit (HCT), and reticulocyte levels compared to the NS group. Hemoglobin (HGB) level showed an increasing tendency but did not reach statistical significance (*p* = 0.09) (Figure 1B).

In addition, we examined whether WOL treatment had any adverse effects on the liver or kidney functions of the mice. The liver enzymes AST and ALT were measured to assess liver function, while creatinine levels were used as an indicator of kidney function. The results revealed no significant differences in AST, ALT, and creatinine levels between the WOL-treated group and the NS group (Figure 1C), indicating that WOL had no toxicity on the liver and kidney functions of the mice. Furthermore, we observed no differences in the weight and length of the organs (liver, kidney, and spleen) between the two groups (Figure 1C). Altogether, the WOL-treated mice exhibited enhanced hematological parameters with no signs of toxicity.

#### 3.1.2. WOL Promoted Plasma Iron Level and Splenic Ferroportin Expression in Mice

**Figure 2:**
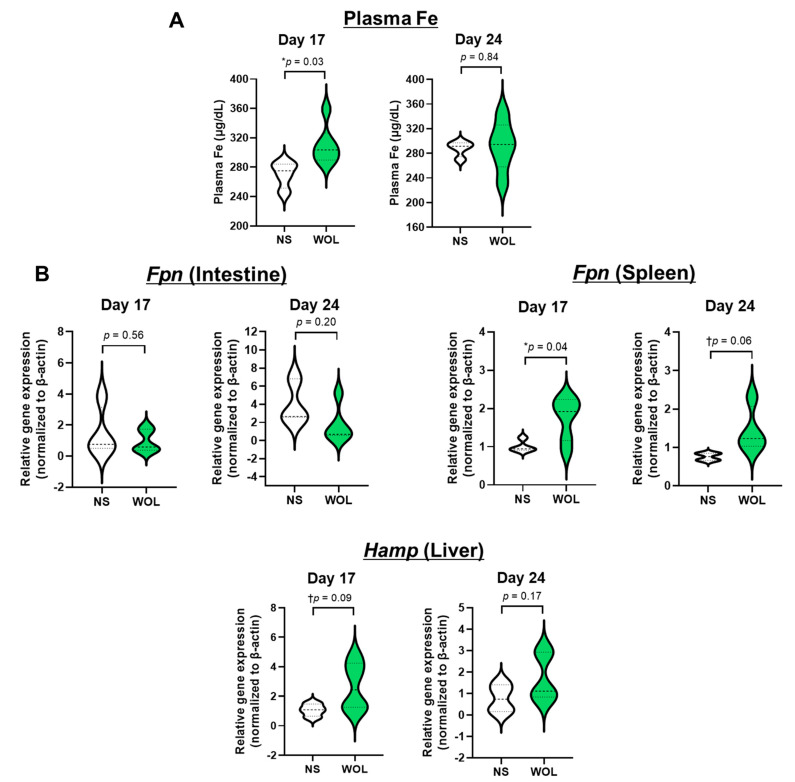
WOL promoted plasma Fe level and splenic *Fpn* expression in mice. The mice were orally administrated with 150 mg/kg bw WOL for 17 and 24 days. (**A**) The plasma iron level and (**B**) gene expressions (*Fpn* in the intestine and spleen and *Hamp* in the liver) were analyzed in the mice. Each value represents the mean ± SE for *n* = 4 (NS) and *n* = 6 (WOL) in each group. Significant difference from NS group at ^†^ *p* < 0.1 and * *p* < 0.05 by unpaired two-tailed Student’s *t*-test. The lines in the middle of the violin chart area refer to the mean value. NS: normal saline-treated control group; WOL: aqueous extract of olive leaves-treated group; Fe: iron; Fpn: ferroportin; Hamp: hepcidin.

To investigate the effects of WOL on iron metabolism in the mice, we measured plasma iron (Fe) levels. The mice treated with WOL for 17 days showed a significant increase in plasma Fe compared to the NS group (Figure 2A).

Furthermore, gene expression analysis revealed that oral administration of WOL led to a significant increase in splenic ferroportin (*Fpn*) expression on Day 17, suggesting a potential role of WOL in regulating cellular iron export in the spleen. However, intestinal *Fpn* expression remained unaffected during this period (Figure 2B). Moreover, the expression of liver hepcidin (*Hamp*), which is involved in iron regulation, exhibited a tendency to increase under the influence of WOL, though it did not reach statistical significance (*p* = 0.09).

#### 3.1.3. WOL Promoted Hif1a Expression in the Kidney of the Mice

**Figure 3:**
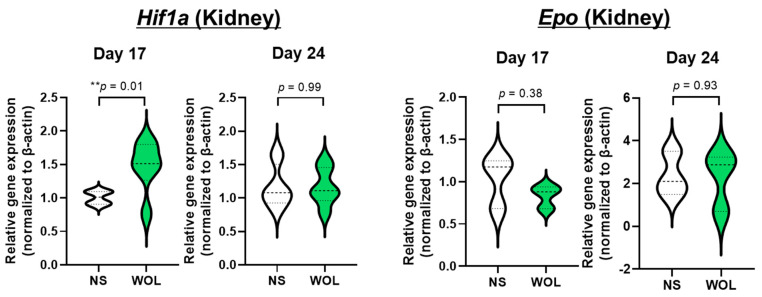
WOL promoted *Hif1A* expression in the mice’s kidneys. The mice were orally administrated with 150 mg/kg bw WOL for 17 and 24 days. Gene expressions (*Hif1a and Epo* in the kidney) were analyzed in RT-qPCR. Each value represents the mean ± SE for *n* = 4 (NS) and *n* = 6 (WOL) in each group. Significant difference from NS group at ** *p* < 0.01 by unpaired two-tailed Student’s *t*-test. The lines in the middle of the violin chart area refer to the mean value. NS: normal saline-treated control group; WOL: aqueous extract of olive leaves-treated group; Hif1a: hypoxia-inducible factor 1 alpha; Epo: erythropoietin.

Oral administration of WOL significantly increased the expression of kidney *Hif1a* on Day 17 compared to the NS group (Figure 3). On the other hand, no effect of WOL was observed in kidney erythropoietin (*Epo*) expression.

### 3.2. Pilot Study

#### WOL Supplementation Significantly Increased RBC Count and Hemoglobin in Healthy Volunteers

A single-arm pilot study was conducted with healthy volunteers to evaluate the feasibility, compliance, and potential benefits of WOL. A total of seven participants received a daily supplementation of 500 mg/kg of OLE for a duration of 8 weeks.

The participants displayed commendable adherence to the WOL treatment throughout the entire 8-week intervention. All seven participants successfully completed the trial, reflecting their high compliance rates. To improve palatability, the formulated powder was encapsulated in HPMC capsules, effectively concealing any specific odor or unpleasant taste associated with the supplement.

**Figure 4:**
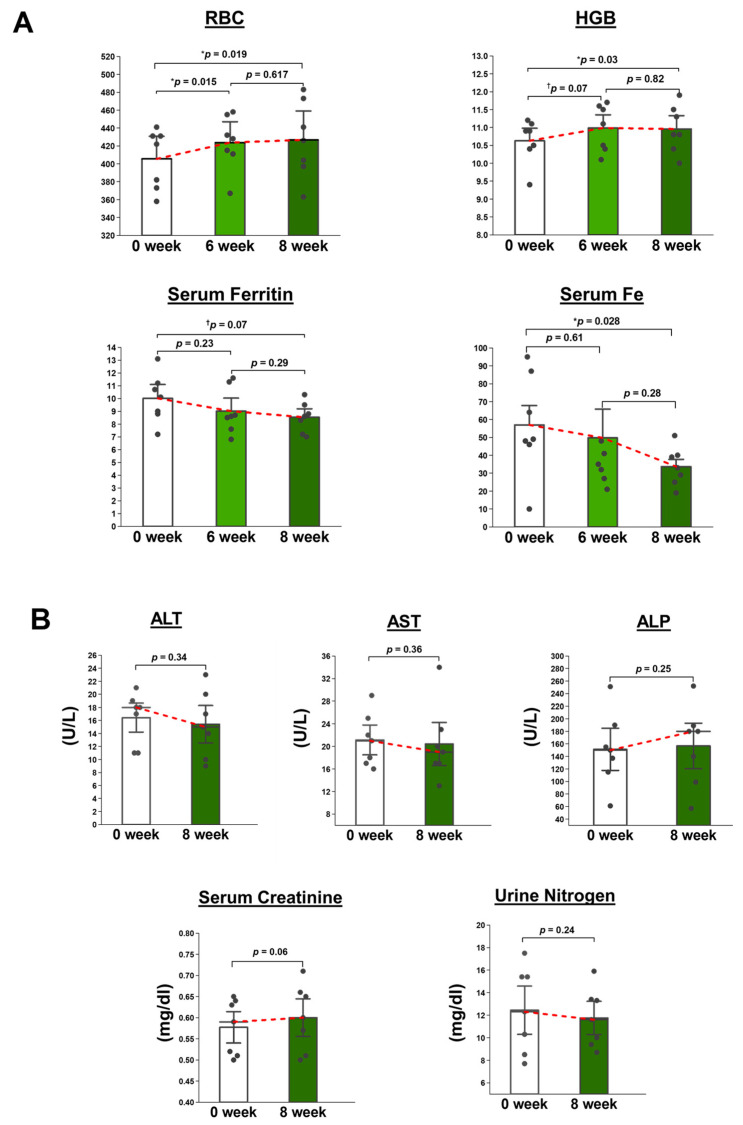
WOL promoted erythropoiesis in healthy volunteers and showed no toxicity. The healthy volunteers were supplemented with 500 mg/kg bw WOL for 8 weeks. (**A**) Hematological parameters (RBC, HBG) and iron metabolism markers (Ferritin and Fe in serum). (**B**) Hepatic dysfunction markers (ALT, AST, and ALP in serum) and renal dysfunction markers (serum creatinine and urine nitrogen). Each value represents the mean ± SE in each group. ^†^ *p* < 0.1, * *p* < 0.05 by one-way repeated measure ANOVA followed by Fisher’s least significant difference (LSD) test. The bar represents the mean value, the error bar represents the SEM, and the red dashed line connects the median values. RBC: red blood cell; HGB: hemoglobin, Fe: iron; ALT: alanine aminotransferase; AST: aspartate aminotransferase; ALP: alkaline phosphatase.

A one-way repeated measure ANOVA was conducted to compare the effect of WOL supplementation on hematological parameters at baseline and 6 weeks and 8 weeks after intervention (Figure 4A). In comparison to the baseline measurements, there was a significant increase (*p <* 0.05) in RBC count observed at both the 6-week and 8-week post-evaluation. HGB exhibited a borderline significant increase (*p <* 0.1) at 6 weeks and a significant increase (*p <* 0.05) after 8 weeks of intervention. Moreover, after 8 weeks of intervention, serum Fe displayed a significant decrease (*p* < 0.05), while serum ferritin demonstrated a borderline significant decrease (*p* < 0.1) in comparison to the baseline levels.

Other parameters, including HCT and platelet count, did not exhibit any significant changes. However, the WBC count was significantly lower at 8 weeks of intervention compared to the 6-week mark (Supplementary Figure S1).

The safety parameters of liver function and renal function tests, including ALT, AST, ALP, serum creatinine, and urine nitrogen, did not demonstrate any significant changes resulting from the 8-week WOL intervention (Figure 4B).

### 3.3. Phytochemical Component Study

**Figure 5:**
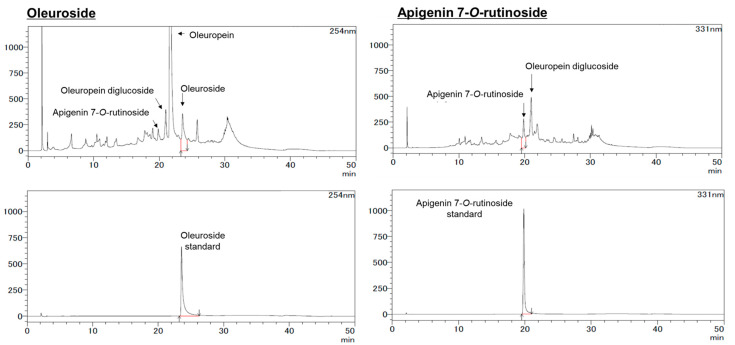
The HPLC chromatograms of the water extract of olive leaf (WOL). WOL contained oleuroside and apigenin-7-*O*-rutinoside in addition to the six components. The samples were analyzed with a water and acetonitrile mixture containing 0.5% acetic acid as the mobile phase and detected at 254 nm or 331 nm. The chromatograms for oleuroside (254 nm) are shown on the left side, while the chromatograms for apigenin-7-*O*-rutinoside (331 nm) are shown on the right side.

HPLC analysis was used to identify and quantify the components in 120 µg/mL WOL in the previous study. The results of previous studies showed that apigenin (0.711 ± 0.003 mg/g dry weight), A7G (1.763 ± 0.030 mg/g dry weight), hydroxytyrosol (3.596 ± 0.013 mg/g dry weight), luteolin (1.588 ± 0.008 mg/g dry weight), L7G (30.590 ± 0.129 mg/g dry weight), and oleuropein (159.250 ± 0.396 mg/g dry weight) in WOL were identified and quantified [22]. Furthermore, in this study, modifications were made to the HPLC conditions to enable the detection of other components. As a consequence, two additional compounds, oleuroside (OS; 23.571 mg/g dry weight) and apigenin-7-*O*-rutinoside (A7R; 12.571 mg/g dry weight) were newly identified. HPLC chromatograms are shown in Figure 5. ESI-MS spectra data for A7R and OS are given in the supplementary file (Supplementary Figures S2 and S3).

### 3.4. Cell Culture Study

#### 3.4.1. WOL Induced Erythroid Lineage Differentiation in K562 Cells, and This Activity Was Attributed to the Synergistic Effects of OP and OS

**Figure 6:**
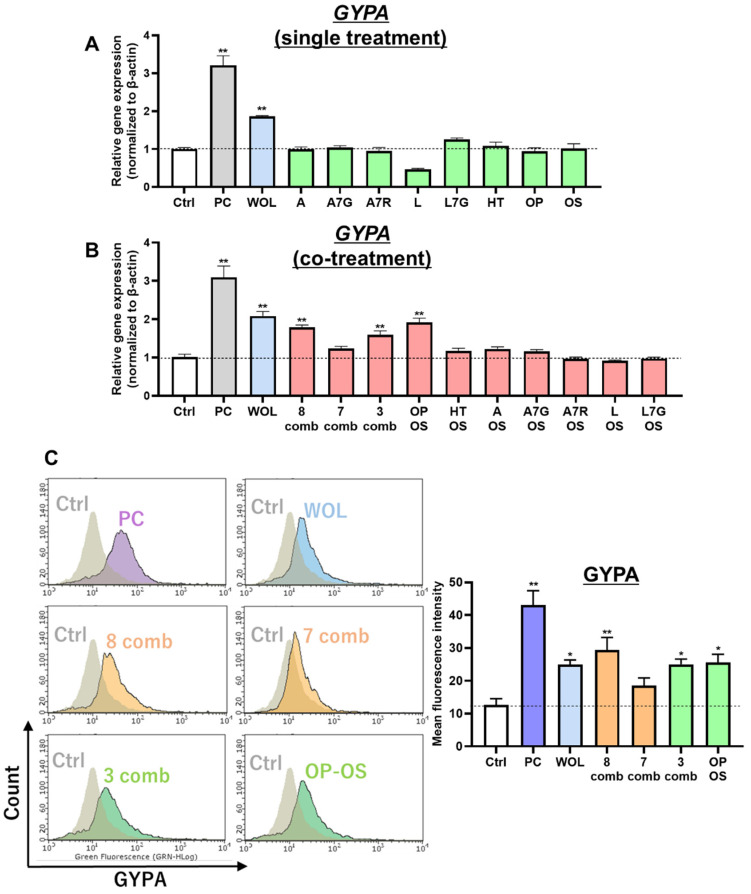
WOL induced erythroid lineage differentiation in K562 cells and this activity was attributed to the synergistic effects of OP and OS. The K562 cells were treated with 120 µg/mL WOL for 6 days. Gene expressions of erythroid differentiation marker GYPA in the cells single-treated (**A**) or co-treated (**B**) in eight components of WOL were examined by real-time PCR. The mRNA expressions were normalized to β-actin as the internal control. Protein expressions of GYPA in the cells co-treated in eight components of WOL were examined by mean fluorescence of flow cytometry (**C**). Each value represents the mean ± SE for *n* = 4. Statistically significant difference from the Ctrl at * *p* < 0.05 and ** *p* < 0.01 by one-way ANOVA followed by Tukey’s post hoc test. GYPA: glycophorin A; Ctrl: control; PC: positive control; WOL: aqueous extract of olive leaves; A: apigenin, A7G: apigenin 7-glucoside; A7R: apigenin-7-*O*-rutinoside; HT: hydroxytyrosol; L: luteolin; L7G: luteolin 7-glucoside; OP: oleuropein; OS: oleuroside; comb: combination.

We attempted to identify the active components of WOL with erythroid differentiation-inducing activity. WOL significantly increased the gene expression of the erythroid differentiation marker *GYPA* in K562 cells compared to the control (Ctrl) (Figure 6A–C). The K562 cells were treated with the eight components- A, A7G, A7R, HT, L, L7G, OP, and OS.

Single treatment of eight components in WOL did not increase *GYPA* expression in the cells (Figure 6A). Next, each combination of the eight components was co-treated into K562 cells, and co-treatment of all eight components significantly increased *GYPA* expression (Figure 6B). On the other hand, the seven-component combination without OS showed no change in *GYPA* expression (Figure 6B). Three components (HT, OP, and OS) and two components (OP and OS), including OS, showed an increase in *GYPA* expression (Figure 6B). Co-treatment of OS with other components (OS + HT, A, A7G, A7R, L, or L7G) did not increase *GYPA* expression (Figure 6B). Flow cytometry was employed to analyze GYPA protein levels (mean fluorescence intensity) on the surface of K562 cells. The results demonstrated that treatment with WOL, all eight components, a combination of three components (HT, OP, and OS), and a combination of two components (OP and OS) all led to an increase in GYPA expression. These findings are consistent with the results obtained from the gene expression level analysis (Figure 6C).

#### 3.4.2. The Effect of Co-Treatment of OP with OS on the Induction of Erythroid Differentiation Increased in an OS Concentration-Dependent Manner

**Figure 7:**
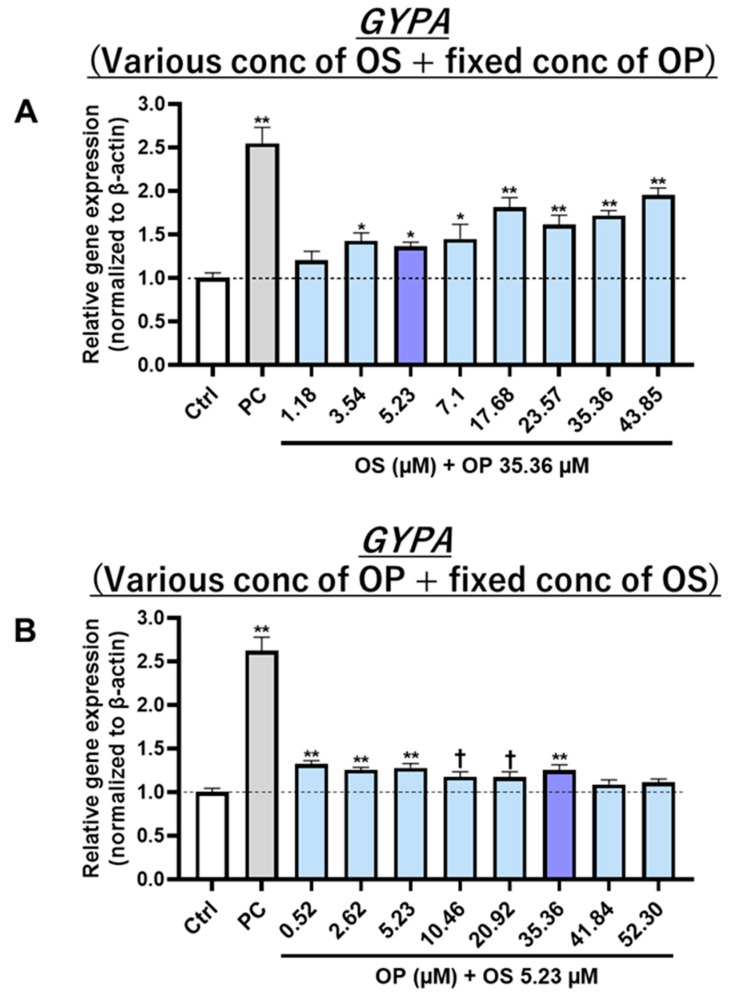
The effect of the co-treatment of OP with OS on the induction of erythroid differentiation increased in an OS concentration-dependent manner. The K562 cells were co-treated with OP and OS at various concentrations for 6 days. Gene expressions of erythroid differentiation marker GYPA in the cells treated with varied OS concentrations and fixed OP concentration (**A**) or varied OP concentrations and fixed OS concentration (**B**) were examined by real-time PCR. The mRNA expressions were normalized to β-actin as the internal control. Each value represents the mean ± SE for *n* = 4. Statistically significant difference from the Ctrl at ^†^ *p* < 0.1, * *p* < 0.05, and ** *p* < 0.01 by one-way ANOVA followed by Tukey’s post hoc test.

To investigate the potential of the co-treatment of OP and OS in inducing erythroid differentiation, K562 cells were co-treated with various concentrations of OP and OS and measured *GYPA* gene expression levels. The same OP concentration as WOL was fixed and the OS concentration was varied and co-treated into K562 cells (OP: 35.36 µM, OS: various concentrations). The results showed that co-treatment of OP and OS increased *GYPA* expression in an OS concentration-dependent manner (Figure 7A). Conversely, the same OS concentration as WOL was fixed, and the OP concentration was varied in the co-treatment (OP: various concentrations, OS: 5.23 µM). As a result, the increase in *GYPA* expression was constant, and the increase in *GYPA* expression disappeared when OP was above 41.84 µM (Figure 7B).

#### 3.4.3. Viability and Proliferation of K562 Cells Treated by WOL and Its Components

The viability and proliferation rates in K562 cells treated with WOL and its components showed similar trends, with all eight components and three-component combinations reducing viability and proliferation compared to the Ctrl (Supplementary Figure S4A,B). Other combinations, including OP-OS, had no effect on either viability or proliferation.

## 4. Discussion

This present study has provided novel evidence, for the first time, of WOL’s positive influence on hematological parameters in mice and healthy volunteers. Additionally, our investigation has successfully identified the active components within WOL responsible for inducing erythroid differentiation.

After 24 days of orally administering WOL to healthy mice, hematological metrics like RBC, HCT, HGB, and reticulocytes showed an increase. Among these metrics, all except for HGB displayed statistically significant changes. In healthy volunteers, WOL supplementation significantly increased RBC count and HGB. This finding is consistent with our previous clinical trial study that reported the beneficial effects of the long-term consumption of olive leaf tea on hematological parameters in humans [18].

The results of plasma biologicals and organ weights suggest that WOL administration was not toxic to the mice, and similar results were obtained from serum analysis in healthy volunteers. The release of intracellular enzymes such as AST, ALT, and ALP is a prominent indicator of hepatocellular injury [25]. Elevated serum creatinine is considered an indicator of renal toxicity [26]. Changes in liver and kidney weight (gain or loss) are important indicators of organ damage following exposure to toxic substances [27,28]. Additionally, splenomegaly, characterized by spleen enlargement, can arise due to hematologic abnormalities or infections [29]. The administration of WOL to healthy mice did not result in any changes to the weights of the liver, kidney, or spleen. Additionally, parameters indicating liver and kidney toxicity, including AST, ALT, and creatinine, remained unchanged without significant alterations in both WOL-supplemented volunteers and WOL-treated mice.

The response of iron metabolism-promoting mechanisms to WOL was also observed in mice. WOL has been found to release recycling iron into the blood by increasing splenic *Fpn* expression in mice, thereby supplying the iron necessary for erythropoiesis.

Hepcidin, a hepatic peptide, has been identified as a systemic iron-regulating hormone that regulates intestinal iron absorption, plasma iron concentration, and tissue iron distribution by inducing degradation of its receptor, the cellular iron exporter FPN [30]. In the intestine, *Fpn* controls iron absorption from food intake, while in the spleen, *Fpn* regulates iron recycling via phagocytosis of senescent erythrocytes by macrophages [31]. In this study, there was increased *Hamp* expression in the mice. This may be a function of controlling the release of recycling iron from the spleen by suppressing splenic *Fpn* expression to maintain iron homeostasis in the body.

In addition, WOL increased renal *Hif1a* expression, which is expected to induce erythroid differentiation. However, other findings have reported that the GATA transcription factor, which is essential for erythroid differentiation, binds specifically to the GATA element of the *Epo* gene promoter and negatively regulates *Epo* gene expression [32]. In our previous study, WOL also markedly upregulated *GATA* gene expression in human bone marrow-derived hematopoietic stem cells (HSCs) [22]. This suggests that WOL may regulate the negative feedback of EPO expression via elevated GATA expression. The results also suggest that WOL may promote erythroid differentiation without EPO. A previous study confirmed that treatment of human bone marrow-derived HSCs with WOL in the absence of EPO resulted in differentiation into erythroid lineage cells [22]. This suggests that WOL induces erythropoiesis in an EPO-independent manner. Additionally, changes in plasma iron and renal *Hif1a* between 17 and 24 days may have been negative feedback of HAMP and HIF degradation under normoxia to maintain homeostasis in the blood environment [33,34].

Previous studies have investigated various components in olive leaf inducing erythroid differentiation of K562 cells, such as apigenin 7-O-beta-D-glucoside (apigetrin) [20]. In the present study, it was initially determined that treatment with 120 µg/mL WOL induced the differentiation of K562 cells towards the erythroid linage, which represented a significant increase in the expression of the erythroid marker GYPA. When investigating the active components, it was ascertained that this inducing effect is not from any single component in WOL but rather from a combination. The combination of all eight main components showed a similar effect to WOL, while when OS was removed from the combination, the increased expression of GYPA could not be confirmed. In addition, experiments involving the combination of OS with each of the other seven components ultimately provided conclusive evidence that the erythroid differentiation-inducing effect of WOL resulted from the combination of OP and OS (Figure S4A). This effect was also dose-dependent for OS under fixed OP concentrations (Figure S4B). Further research is needed to clarify the mechanisms of OP and OS interaction. Additionally, having detected the presence of apigenin 7-O-rutinoside and OS compounds within WOL, it becomes worthwhile to investigate their glycosidic bond configuration in future studies, with a specific focus on distinguishing between alpha and beta linkages.

Most importantly, our pilot study demonstrated that short-term WOL intake has the potential to improve RBC count and HB in healthy volunteers without any adverse effects on liver and kidney functions. Additionally, WOL was found to be well-accepted among the volunteers. In the future, it is warranted to conduct a double-blind, randomized, controlled clinical trial with a longer duration and a larger sample size to validate and confirm the hematinic effects of WOL.

## 5. Conclusions

This study encompassed both human and mouse investigations, which collectively demonstrated the hematological beneficial effects of WOL without inducing any toxic effects under healthy conditions. The underlying mechanism responsible for this erythropoiesis-promoting effect is believed to involve the facilitation of recycling iron release from the spleen and the absorption of dietary iron in the intestine, thereby supplying the iron essential for erythropoiesis. Furthermore, cell experiments have conclusively confirmed that the active components responsible for inducing erythroid differentiation are the combined effects of oleuropein and oleuroside, working synergistically to achieve this effect. Altogether, our study presents evidence of the hematinic potential of WOL in in vivo studies, opening up exciting possibilities for future applications in preventing or treating anemia.

## 6. Patents

The data reported in this article have been used to apply for a patent under Japanese Patent Application No. 2021-123559 (Registration No. 6540169; URL: https://www.j-platpat.inpit.go.jp/c1800/TR/JP-2021-123559/CEA1A080BBD09810B45DB7FDE978144AD6586E981EA2ACDF5ADA5E4FB107D301/40/en) (Registered on 1 April 2022).

## Data Availability

The data that support the findings of this study are available in this article and in the supporting information of this article.

## Supplementary Materials

The following supporting information can be downloaded at: https://www.mdpi.com/article/10.3390/nu15194095/s1, Figure S1: Effects of WOL on hematocrit level, WBC count, and platelet count in human volunteers; Figure S2: ESI-MS spectra of authentic apigenin-7-*O*-rutinoside (top) and its component in WOL (bottom); Figure S3: ESI-MS spectra of authentic oleuroside (top) and its component in WOL (bottom); Figure S4: Viability and Proliferation of K562 cells treated by WOL and its components; Supplementary Table S1: Content of WOL capsule.
