# Supplementary material for: Hematinic Potential of Olive Leaf Extract: Evidence from an In Vivo Study in Mice and a Pilot Study in Healthy Human Volunteers
Source: Nutrients. 2023 Sep 22;15(19):4095. doi: 10.3390/nu15194095 (PMC10574213; doi:10.3390/nu15194095)
Supplement: Supplementary file 1 [file nutrients-15-04095-s001.zip › nutrients-2579284-supplementary.pdf]

nutrients

MDPI

Supplementary Data

Hematinic Potential of Olive Leaf Extract: Evidence from an in vivo study in mice and a pilot study in healthy human volunteers

Shinji Kondo [1,†], Farhana Ferdousi [1,2,†], Jinchang Zhao [3], Sofya Suidasari [4], Miki Yokozawa [4], Ken Yamauchi [4], Ken-ichi Tominaga [5] and Hiroko Isoda [1,2,5,*]

[1] Alliance for Research on the Mediterranean and North Africa (ARENA), University of Tsukuba, Tsukuba 305-8572, Japan; kondo.shinji.ga@u.tsukuba.ac.jp (S.K.); ferdousi.farhana.fn@u.tsukuba.ac.jp (F.F.)
[2] Faculty of Life and Environmental Sciences, University of Tsukuba, Tsukuba 305-8575, Japan
[3] Graduate School of Life and Environmental Science, University of Tsukuba, Tsukuba 305-8572, Japan; jinchang_zhao@outlook.com
[4] Nutrition Act Co., Ltd., Ginza, Tokyo 104-0061, Japan; sofya-s@n-act.co.jp (S.S.); m-yokozawa@n-act.co.jp (M.Y.); k-yamauchi@n-act.co.jp (K.Y.)
[5] Open Innovation Laboratory for Food and Medicinal Resource Engineering (FoodMed-OIL), National Institute of Advanced Industrial Science and Technology (AIST), Tsukuba 305-8577, Japan; k-tominaga@aist.go.jp
* Correspondence: isoda.hiroko.ga@u.tsukuba.ac.jp; Tel.: +81-029-853-5775
† These authors contributed equally to this work.

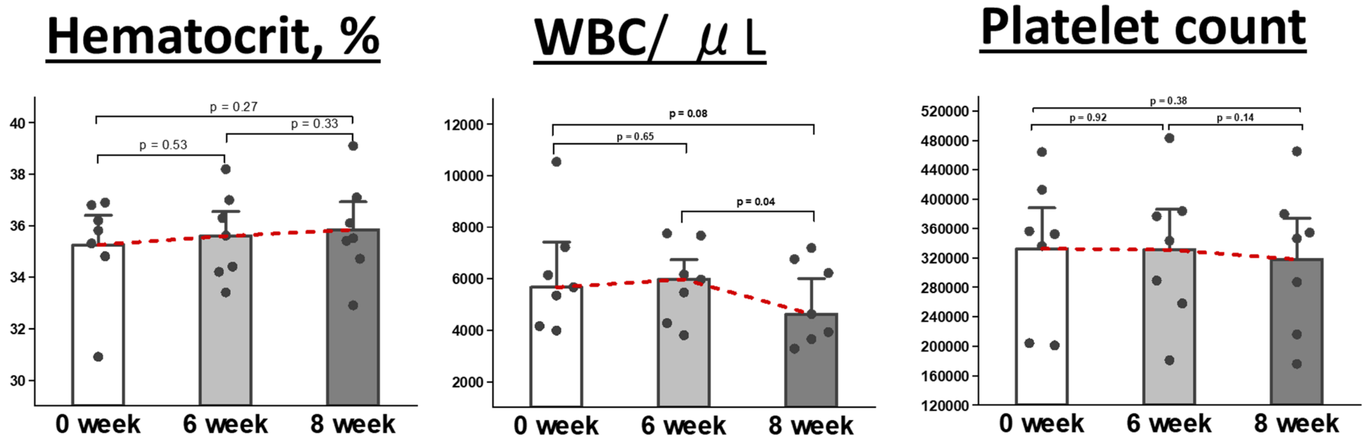

Figure S1. Effects of WOL on hematocrit level, WBC count, and platelet count in human volunteers.

P-values from one-way repeated measures ANOVA followed by Fisher's least significant difference (LSD) test. The bar represents the mean value, the error bar represents the SEM, and the red dash line connects the median values.

Table S1. Content of WOL capsule.

Raw material name	Test food (per capsule)
Olive leaf extract powder	125.00mg
Starch hydrolysate	236.90mg
Fine silicon dioxide	3.85mg
Calcium stearate	19.25mg
Membrane HPMC hard capsule (White)	70mg
Total	455.00mg

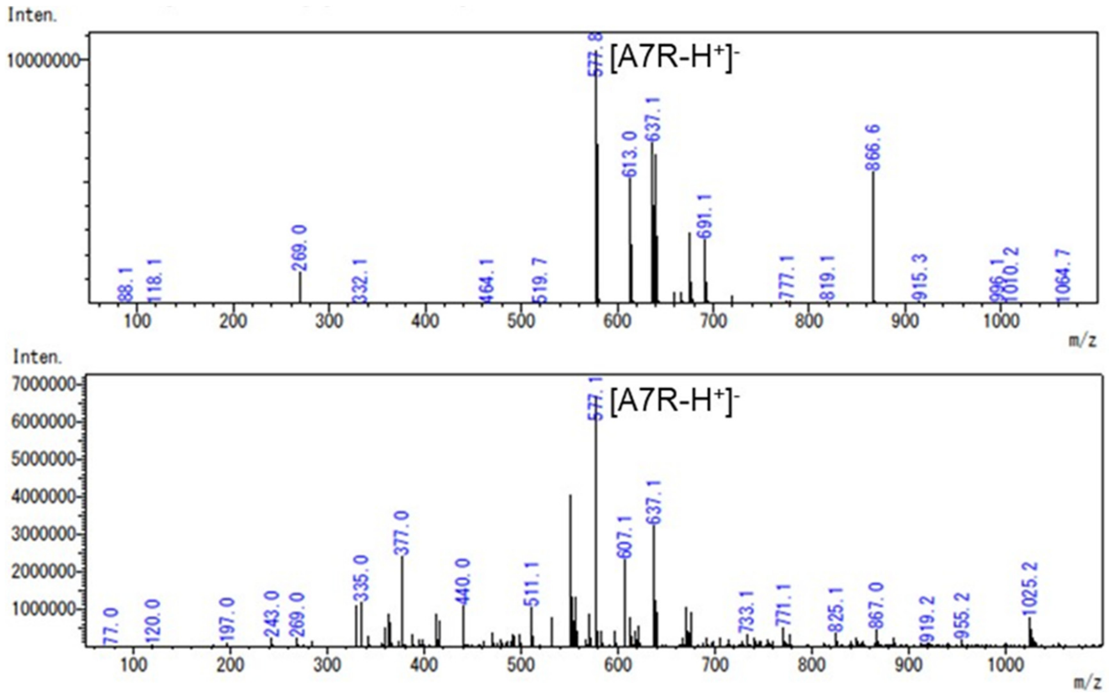

Figure S2. ESI-MS spectra of authentic apigenin-7-*O*-rutinoside (top) and its component in WOL (bottom).

Apparatus: Shimadzu LC-2020, MS mode: ESI-negative, interface voltage: -3.5 kV, nebulizer gas: N2 (1.5 L/min), drying gas: N2 (15 L/min), column: TSKgel ODS-100V 3 μm Φ2.0 mm 150 mm, column temp.: 40 °C, eluent: 0.5% CH3COOH / CH3CN, gradient: B 0 min 5 %, 5 min 15 %, 25 min 30 %, and 35 min 95 %.

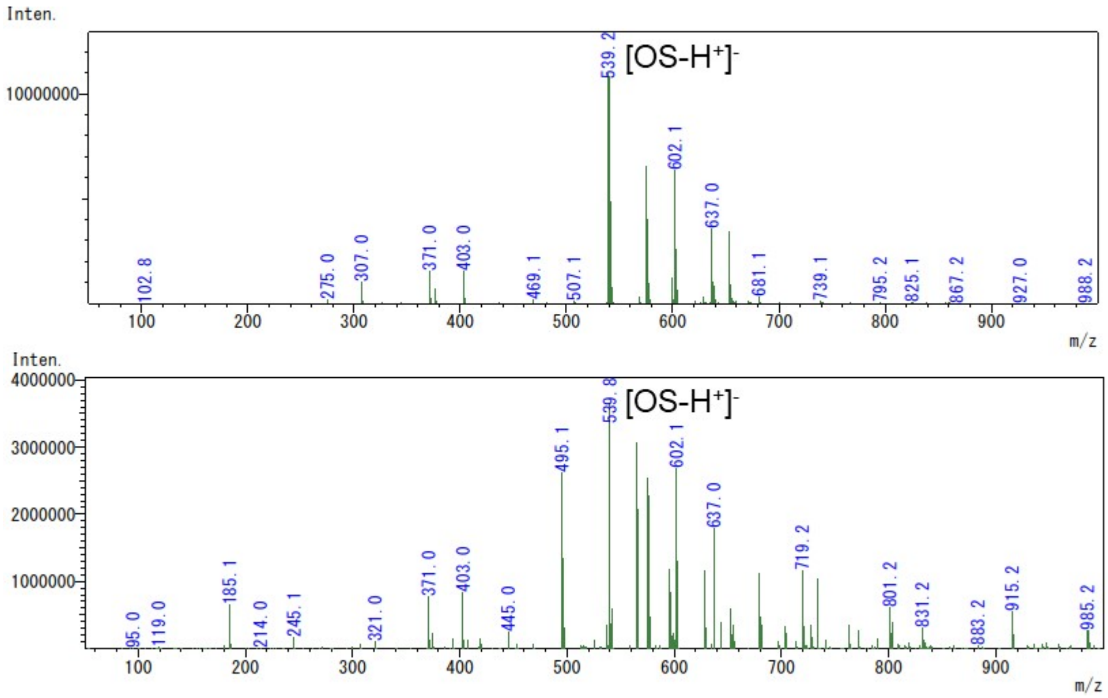

Figure S3. ESI-MS spectra of authentic oleuroside (top) and its component in WOL (bottom).
Apparatus: Shimadzu LC-2020, MS mode: ESI-negative, interface voltage: -3.5 kV, nebulizer gas: N2 (1.5 L/min), drying gas: N2 (15 L/min), column: TSKgel ODS-100V 3 μm Φ2.0 mm   150 mm, column temp.: 40 °C, eluent: 0.5% CH3COOH / CH3CN, gradient: B 0 min 5 %, 5 min 15 %, 25 min 30 %, and 35 min 95 %.

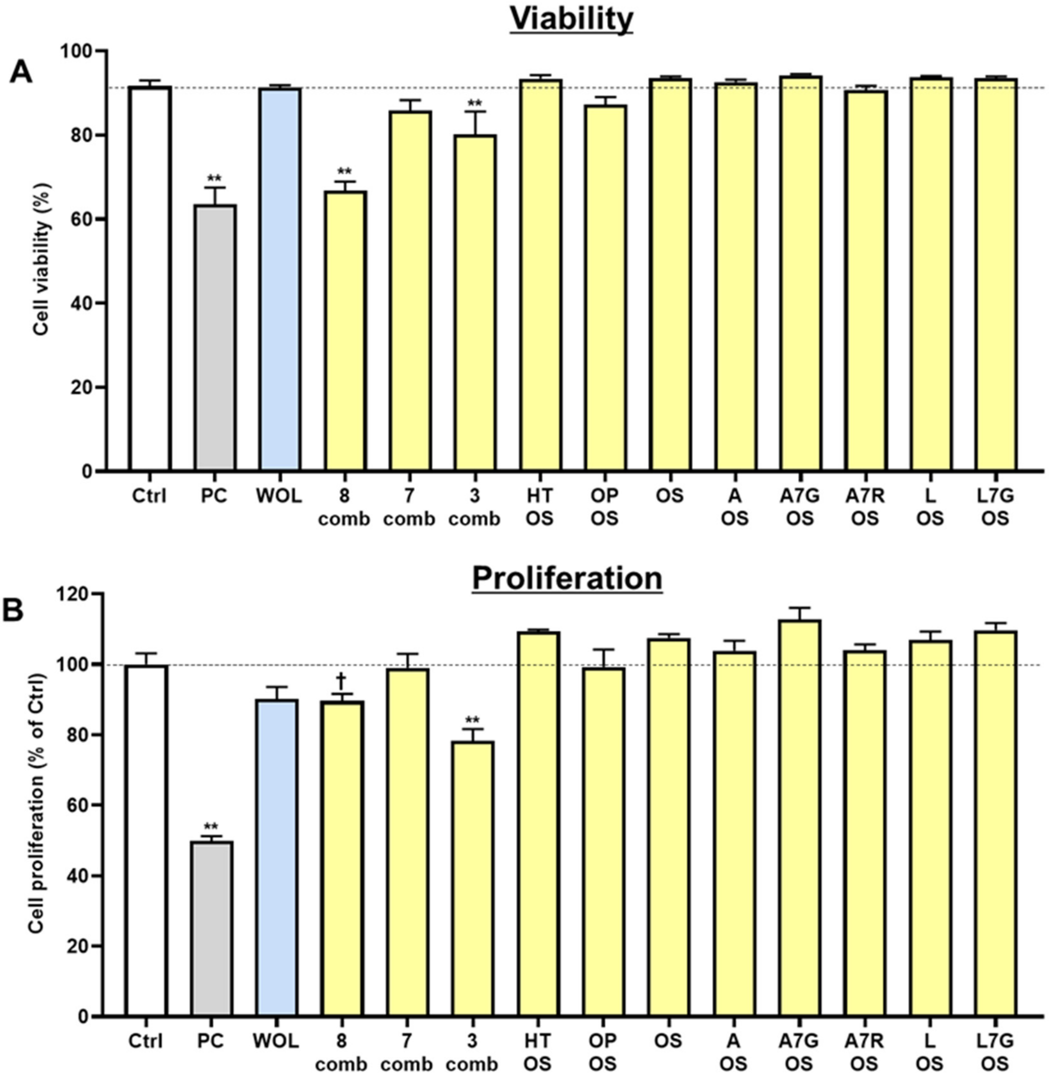

Figure S4. Viability and Proliferation of K562 cells treated by WOL and its components.
The K562 cells were treated with 120 μg/mL WOL for 6 days. The viability was detected via viaCount (A) and the proliferation was detected by the MTT assay (B). Each value represents the mean ± SE for n = 4 on a 6-well plate. Significant difference from the Ctrl at $^{\dagger}P < 0.1$, $^{*}P < 0.05$, and $^{**}P < 0.01$ by one-way ANOVA followed by Tukey's post hoc test.
